# Ebola virus mucin-like glycoprotein (Emuc) induces remarkable acute inflammation and tissue injury: evidence for Emuc pathogenicity *in vivo*

**DOI:** 10.1007/s13238-017-0471-x

**Published:** 2017-09-27

**Authors:** Yun-Jia Ning, Zhenyu Kang, Jingjun Xing, Yuan-Qin Min, Dan Liu, Kuan Feng, Manli Wang, Fei Deng, Yiwu Zhou, Zhihong Hu, Hualin Wang

**Affiliations:** 1 State Key Laboratory of Virology, Wuhan Institute of Virology, Chinese Academy of Sciences, Wuhan, 430071 China; 2 University of Chinese Academy of Sciences, Beijing, 100049 China; 3 Department of Forensic Medicine, Tongji Medical College, Huazhong University of Science and Technology, Wuhan, 430030 China; 4 School of Medicine, Wuhan University of Science and Technology, Wuhan, 430081 China

**Dear Editor,**

Ebola virus (EBOV) is one of the most virulent pathogens to humans. Recently, the largest-ever outbreak of Ebola virus disease (EVD) in West Africa, 2013–2016, resulted in the unprecedented damage to human health and social economy. However, there is currently no licensed vaccine or antiviral available against EVD. Clinically, EBOV infection can result in exaggerated inflammatory responses and multiorgan damage, although the pathogenesis (including the inflammatory pathogenesis) and the viral virulence factor(s) involved in the excessive inflammation induction and viral pathogenicity are largely unclear. *In vitro* studies have suggested that the envelope glycoprotein (GP_1,2_) of EBOV mediating virus entry may also directly contribute to the viral pathogenesis, as cell surface expression of GP_1,2_ can induce adherent cell rounding and detachment and sterically mask some cell surface molecules, such as β1-integrin (reviewed in Ning et al., 2017). GP_1,2_ is composed of dimerized subunits GP1 and GP2, locating on the cell or virion membrane; an additional notable feature of GP_1,2_ is that it contains a heavily O-glycosylated mucin-like region within GP1. The functional mapping of GP_1,2_-induced cellular morphological changes showed that both the mucin-like region and cell surface expression by a transmembrane domain (TMD) are required (Ning et al., 2017; Francica et al., 2009; Takada et al., 2000; Yang et al., 2000; Chan et al., 2000), suggesting a role of the surface expressed EBOV mucin-like glycoprotein (Emuc) in the GP_1,2_ effect. However, the biological effects of the glycoproteins have not been investigated systematically *in vitro* and *in vivo*, and there is no direct evidence *in vivo* available for the potential pathogenicity of the glycoproteins.

To investigate the potential pathogenic effects of Emuc both *in vitro* and *in vivo*, we designed an experiment of adenovirus-mediated gene delivery to cultured cells and mice (Fig. 1A). Adenoviruses can infect a broad range of mammalian cells and tissues with the exception of some lymphoid cells. Interestingly, EBOV has a broad cell tropism infecting a wide range of cell types as well, whereas lymphocytes are also resistant to EBOV infection (Feldmann and Geisbert, 2011). For safety concerns, a replication-deficient adenoviral vector (ΔE1/ΔE3) was used in this study (Supplementary Materials and Methods) (He et al., 1998). To achieve the expression and localization similar to the context of viral infection, Emuc was placed into a small membrane protein TVA (a transmembrane glycoprotein product from avian *tv*-*a* genetic locus) to utilize the N-terminal signal peptide (SP) and C-terminal TMD of TVA as previously described (Francica et al., 2009). The Emuc-TVA construct was firstly cloned into a donor vector (pAdT) containing an EGFP expression cassette as a reporter; then the adenoviral vector expressing the Emuc-TVA and EGFP (named ADV-Emuc-TVA) was generated by homologous recombination and virus packaging (Fig. 1A and Supplementary Materials and Methods). Meanwhile, the adenoviral vectors encoding TVA and EGFP (ADV-TVA) or EGFP alone (ADV) were also produced as controls (Fig. 1A). Following PCR and sequencing verification, the expression and subcellular localization of the proteins by these viral vectors were analyzed. As shown in the Western-blot analyses with the antibodies against TVA or Emuc, the cloned genes were efficiently expressed in virus-transduced 293 cells at 24 h post transduction (Fig. 1B); the multiple bands within the TVA and Emuc-TVA lanes likely represent the various glycosylation forms of the proteins (Fig. 1B). Furthermore, in the immunofluorescence assays (IFA) using the TVA or Emuc antibodies, Emuc was obviously detected on the cell membrane (Figs. 1C and S1), indicating that the cell surface expression of Emuc was efficiently achieved. To examine the effects of Emuc *in vitro* using our viral vector transduction system, the morphology of the cultured adherent cells transduced with the viral vectors was monitored by microscopy. As shown in Figure 1D, the transduction of ADV-Emuc-TVA but not the controls, ADV-TVA or ADV, induced noticeable cell rounding and detachment. Furthermore, flow cytometry showed that Emuc expression significantly decreases the surface immunostaining of β1-integrin and human leukocyte antigen class I (HLA-I) (Fig. 1E), indicating the sterical shielding of the cell surface molecules by the extensively glycosylated Emuc. These data confirm the effects of Emuc observed in transient transfection assays (Francica et al., 2009) and suggest that the viral vector efficiently mediated gene expression and functioning by transduction to the cultured cells.

**Figure 1:**
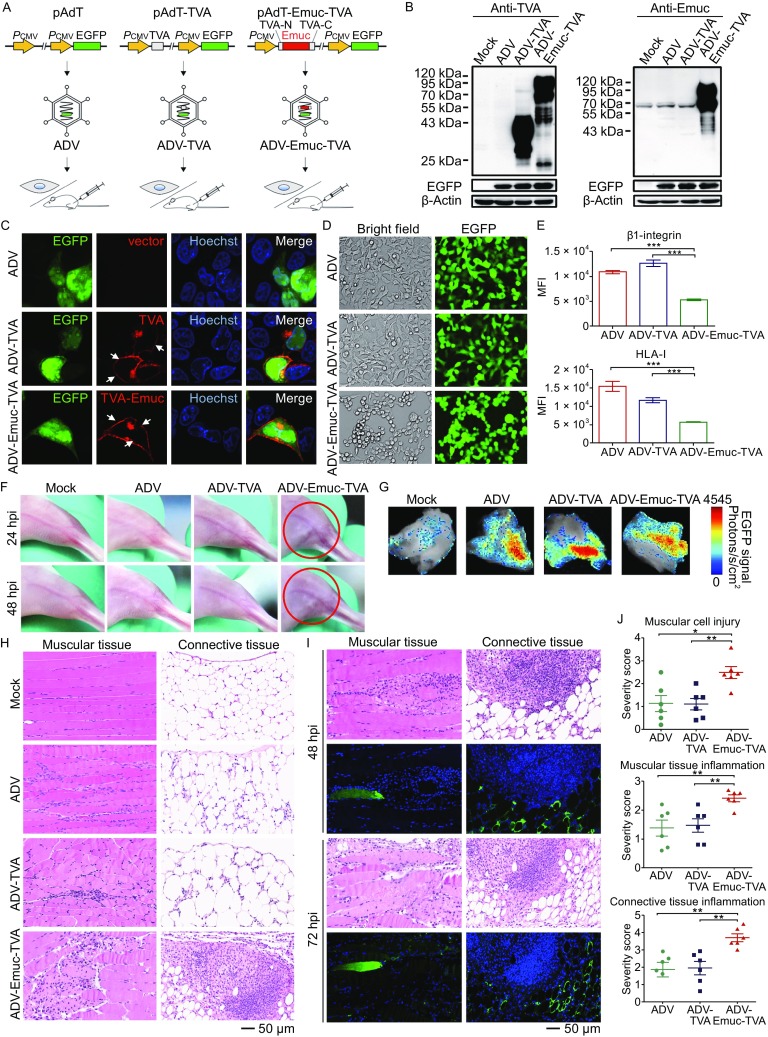
**Adenoviral vector-mediated gene delivery and the potential pathogenicity analyses of EBOV mucin-like glycoprotein (Emuc)** ***in vitro*** **and** ***in vivo***. (A) Technological process of the present research. Firstly, the cassettes encoding the small cell membrane protein TVA or the fused Emuc-TVA were respectively constructed into the donor plasmid pAdT which contains an EGFP expression cassette as the reporter. Adenoviral vectors were then generated by homologous recombinant in bacteria and packaging in HEK293 cells. After identification and purification, the recombinant viral vectors were utilized in the following gene transfer to cultured cells *in vitro* and mouse muscles *in vivo*. (B) Identification of the expression of the cloned genes. HEK293 cells were mock infected or infected by the recombinant adenoviruses ADV-Emuc-TVA, ADV-TVA, or ADV. At 24 hpi, cell lysates were analyzed by Western-blot with the anti-TVA or anti-Emuc antibodies, respectively. EGFP expression was detected for monitoring the efficient and comparable transduction; β-actin was included for sample loading control. The detection was repeated for at least three times with similar results. The multiple bands likely represent the various glycosylation forms of the proteins. (C) Protein subcellular localization. Vero cells infected with the indicated recombinant adenoviruses were fixed and permeabilized at 24 hpi for IFA using the anti-TVA antibody and the localization of target proteins was then visualized under confocal microscope. White arrows indicate the substantial cell membrane localization. See also Fig. S1. (D) Emuc induces rounding and detachment of cultured adherent cells. Adherent HEK293A cells were infected by the indicated viral vectors. At 24 hpi, viral infection (as indicated by EGFP expression) and cellular morphological changes were monitored by a Nikon microscope. A representative result from at least three-time independent experiments was shown. (E) HEK293A cells infected with the indicated viruses were fixed at 36 hpi for surface immunostaining of the cell membrance molecules, β1-integrin or HLA-I, using the corresponding antibodies respectively. Fluorescence signals of the surface molecules were detected by flow cytometry and mean fluorescence intensity (MFI) was analyzed. Graphs show mean ± SD, *n* = 3. ***, *P* < 0.001. (F) BALB/c mice were divided into 4 groups randomly and mock infected or infected with the indicated viruses by intramuscular injection (i.m). Clinical feature then was monitored daily. Circles indicate redness and inextensibility of the hind limb muscles at 24 and 48 hpi, respectively. (G) The mice infected as indicated were euthanized at 48 or 72 hpi, respectively. EGFP signals of the dissected skeletal muscles were detected by an animal imaging system. Representative samples at 72 hpi were shown. (H) Paraffin sections of the indicated skeletal muscles (72 hpi) were stained with H&E for histopathological analyses. See also Figs. S3 and S4. (I) Continuous slices of the paraffin-embedded sample infected by ADV-Emuc-TVA were subjected to H&E staining and IFA for the analyses of histopathology and Emuc-TVA expression, respectively. (J) Quantification of histopathological changes. Unduplicated images of muscular tissues and connective tissues for each sample (72 hpi) as indicated were chosen, respectively, and the degrees of pathological changes in comparison to mock-infected samples were scored by the standard: 0 = normal; 1 = minimal change; 2 = mild change; 3 = moderate change; 4 = marked change; 5 = severe change. Graphs show mean± SD, *n* = 6. **, *P* <0.01; *, *P* < 0.05. See also Fig. S5

Next, we conducted gene delivery of the adenoviral vectors to BALB/c mice for further exploring the effects of Emuc *in vivo*. The viral vectors (1 × 10^8^ PFU) were respectively injected into the adductor magnus muscles of BALB/c mice with local hair depilated. Intramuscular route was chosen for real-time *in situ* observation; additionally, the infection through broken skins and muscles is likely one of the main EBOV transmission routes (Singh et al., 2015; Zaki et al., 1999). In the following days, the clinical feature was closely monitored. Intriguingly, as early as 24 h post infection (hpi), the mice inoculated with ADV-Emuc-TVA showed piloerection and nervousness and the injected hind leg muscles of most mice (7/9) showed mild redness and contracture (Fig. 1F and data not shown). In the preliminary experiments, we observed that the clinical manifestations could last for days, gradually reduced from 72–96 hpi and were invisible after ~1 week (data not shown). Considering that the viral vectors are replication-deficient, the quick recovery of the mice could be associated with the rapid clearance of the injected viral vectors as well as the degradation of expressed proteins and tissue repair. By contrast, no noticeable clinical signs of illness were observed throughout the infection course in the control groups (Fig. 1F). These observations suggest that Emuc expression specifically caused disease, though temporarily, in the context of local transduction by the replication-deficient adenovirus.

Following the euthanization of mice at 48 hpi (*n* = 3) or 72 hpi (*n* = 6), the injected muscles and controls were dissected for further analyses. Firstly, EGFP signals could be detected in the muscles inoculated with viruses under an animal imaging system (Fig. 1G), manifesting the successful transduction of the adenoviral vectors. Subsequently, the viral infection and transgene expression were further analyzed by immunofluorescence staining of paraffin sections using the antibodies against TVA or EGFP. The expression of TVA and EGFP could be detected in multiple cell types (such as myocytes, fat cells, fibroblasts, undifferentiated mesenchymal cells, etc.) of the muscular tissues and connective tissues (Fig. S2 and data not shown), consistent with the broad host cell range of adenoviral vectors. To analyze the histopathological changes, sections were further stained by hematoxylin and eosin (H&E). Intriguingly, the histopathologic examination revealed that Emuc expression by the ADV-Emuc-TVA transduction resulted in massive inflammatory cell infiltration and notable tissue damage in the muscular tissues and connective tissues in the samples at both 48 and 72 hpi, while the infections of the control viruses by contrast only caused slight confined inflammation (Figs. 1H and S3). On close examination, ADV-Emuc-TVA infection induced the infiltration of numerous inflammatory cells (including lymphocytes, monocytes, and neutrophils) into the muscular tissues and connective tissues, particularly the muscular fibers, endomysium, perimysium, connective tissue matrices, and adipose tissues (Figs. 1H, S3, and S4). Moreover, upon the ADV-Emuc-TVA infection, substantial disintegration and necrosis of muscle fibers were evident, congestion and expansion of blood capillaries in the diseased area could be observed, and hyperplasia of connective tissues accompanied with the inflammatory cell infiltration and small abscess formation were also distinct pathological changes (Fig. S4). Meanwhile, the IFA and H&E analyses of continuous slices showed that in the Emuc-expression positive areas, diffuse inflammation and tissue damage were always notable, and vice versa (Fig. 1I), indicating the close association of Emuc expression and the pathological changes. To further quantify the pathological changes, the degrees of pathological changes of muscular tissues and connective tissues in comparison to mock-infected samples were accordingly scored. The control viruses caused minimal to mild pathological changes which were comparable between the ADV and ADV-TVA groups, reflecting the inevitable host immune response against adenoviral vector transduction; however, ADV-Emuc-TVA induced distinct and much severer pathological changes compared with the control groups, further confirming the specific pathogenicity of Emuc *in vivo* (Figs. 1J and S5). Additionally, according to the severity scores, the pathological changes of the control groups, especially the mild inflammation in the connective tissues, seemed to be further alleviated at 72 hpi, compared to 48 hpi, whereas the severe histopathologic changes of Emuc expression group were more persistent (Figs. 1J and S5). Thus, the differences of the pathological severity degrees between ADV-Emuc-TVA and the control groups were more evident at 72 hpi although the specific pathogenic effects caused by Emuc could be obviously observed at both 48 and 72 hpi. Given this, 72 hpi can be a better time point for evaluating the specific pathogenicity of Emuc in this experimental model. Taken together, these data demonstrate that even in the context of local and transient expression by the replication-deficient adenoviral vector, Emuc can cause remarkable acute inflammation and tissue injury, suggesting that Emuc likely is a notable pathogenic factor of EBOV *in vivo*.

*In vitro*, Emuc expression caused rounding and detachment but not death of cultured adherent cells, and i.e., the morphologically changed cells remained viable (Simmons et al., 2002); however, in mouse muscles, Emuc induced not only cell pathologic changes but also cell death, revealing the distinct physiological virulence of Emuc and also highlighting the importance of *in vivo* investigation. Besides the cytopathic effects (CPE) and tissue damage, inflammation is also a significant pathologic effect mediated by Emuc, reminiscent of the excessive inflammation during EBOV infection in clinic (Feldmann and Geisbert, 2011; Baize et al., 2002; Cilloniz et al., 2011) and the capacity of GP_1,2_ and shed GP (both containing Emuc) to activate inflammatory reaction of immune cells *in vitro* (Ning et al., 2017). As shown in Figure 1I, many muscle fibers which seemed not to be infected by ADV-Emuc-TVA but were infiltrated by the inflammatory cells were damaged as well, suggesting that Emuc-mediated inflammation also plays important roles in the cell and tissue injury. Taken together, we consider that the tissue lesion could be mediated directly by the cytotoxicity of Emuc and indirectly by the host inflammatory responses triggered by Emuc although it remains undefined to what extents the two effects of Emuc, respectively, contribute to the tissue damage. Additionally, tissue injury and inflammation can trigger and promote each other under the physiological conditions. These effects likely combinedly contribute to the Emuc pathogenic process *in vivo* (Fig. S6).

As a highly pathogenic virus, EBOV is classified as a biosafety level-4 pathogen, and the manipulations of viral infections are strictly limited by the special containment facilities. As demonstrated here, *in vivo* gene transfer by adenoviral vectors may represent a safe and convenient experimental model which is especially valuable in the physiological function elucidation of the individual virulent proteins encoded by highly dangerous pathogens including EBOV and the development of antivirals specifically targeting the certain virulent proteins such as Emuc identified in this study. The full-length GP of EBOV has been generally used as the immunogen in vaccine researches (Ohimain, 2016) although Emuc is an unnecessary part for GP1,2-mediated entry and is highly variable in amino acid sequence across EBOV strains (Jeffers et al., 2002). Given the virulence of Emuc as shown here, it will be interesting to investigate whether Emuc-deleted GP is a safer and better immunogen. In summary, the present study demonstrated that Emuc can not only induce morphological change of adherent cells *in vitro* but also distinct cell and tissue damage and acute inflammation in mouse muscles, revealing and characterizing the Emuc pathogenicity both *in vitro* and *in vivo*. These findings provide direct evidence of the Emuc pathogenicity *in vivo* for the first time and also critical clues on EBOV pathogenesis and particularly the inflammatory pathogenesis.

## Electronic supplementary material

Below is the link to the electronic supplementary material.

Supplementary material 1 (PDF 780 kb)

Supplementary material 2 (PDF 293 kb)
