# Supplementary material for: Ebola virus mucin-like glycoprotein (Emuc) induces remarkable acute inflammation and tissue injury: evidence for Emuc pathogenicity in vivo
Source: Protein Cell. 2017 Sep 27;9(4):389–93. doi: 10.1007/s13238-017-0471-x (PMC5876185; doi:10.1007/s13238-017-0471-x)
Supplement: Supplementary file 1 — Supplementary material 1 (PDF 780 kb) [file 13238_2017_471_MOESM1_ESM.pdf]

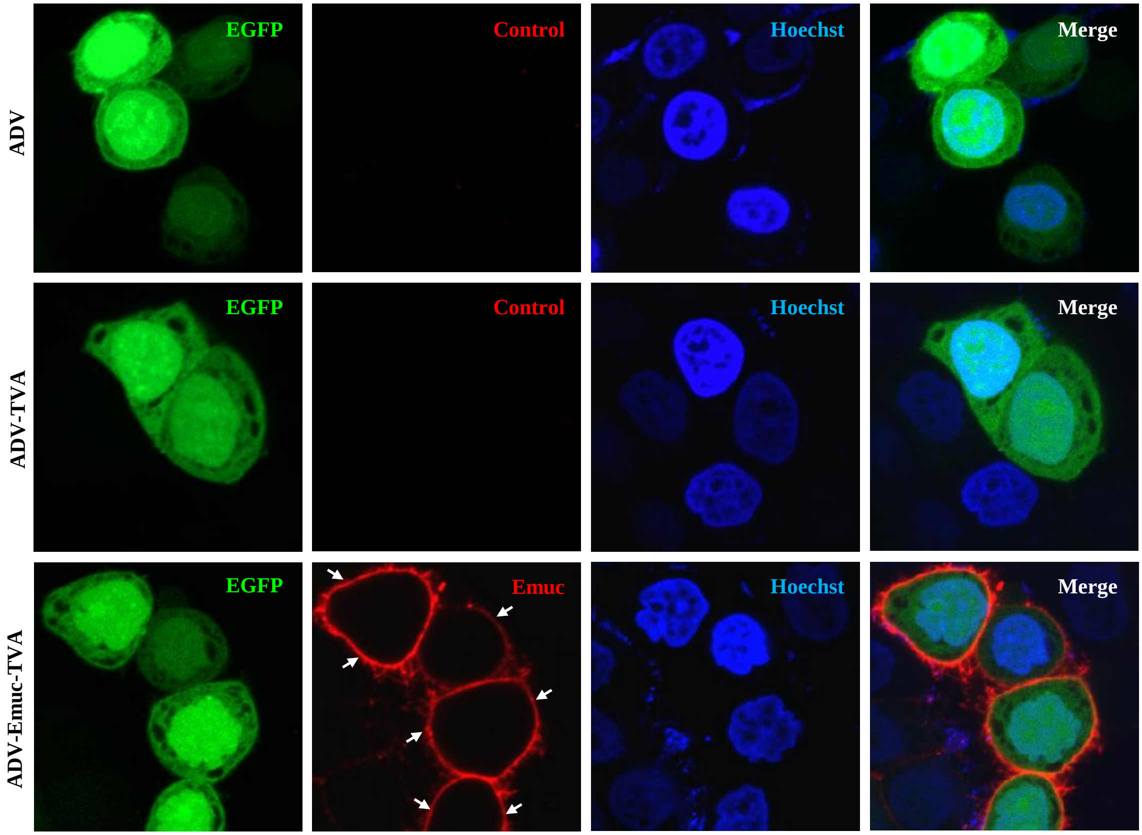

Supplementary Figure S1. Cell surface expression of EBOV mucin-like glycoprotein (Emuc). Vero cells were infected and fixed at 24 hpi as described in Figure 1C but with the exception that these cells were not permeabilized. IFA was then performed with the anti-Emuc antibody. White arrows indicate the specific cell surface localization of Emuc. Nuclei stained with Hoechst are shown in blue. See also Figure 1C.

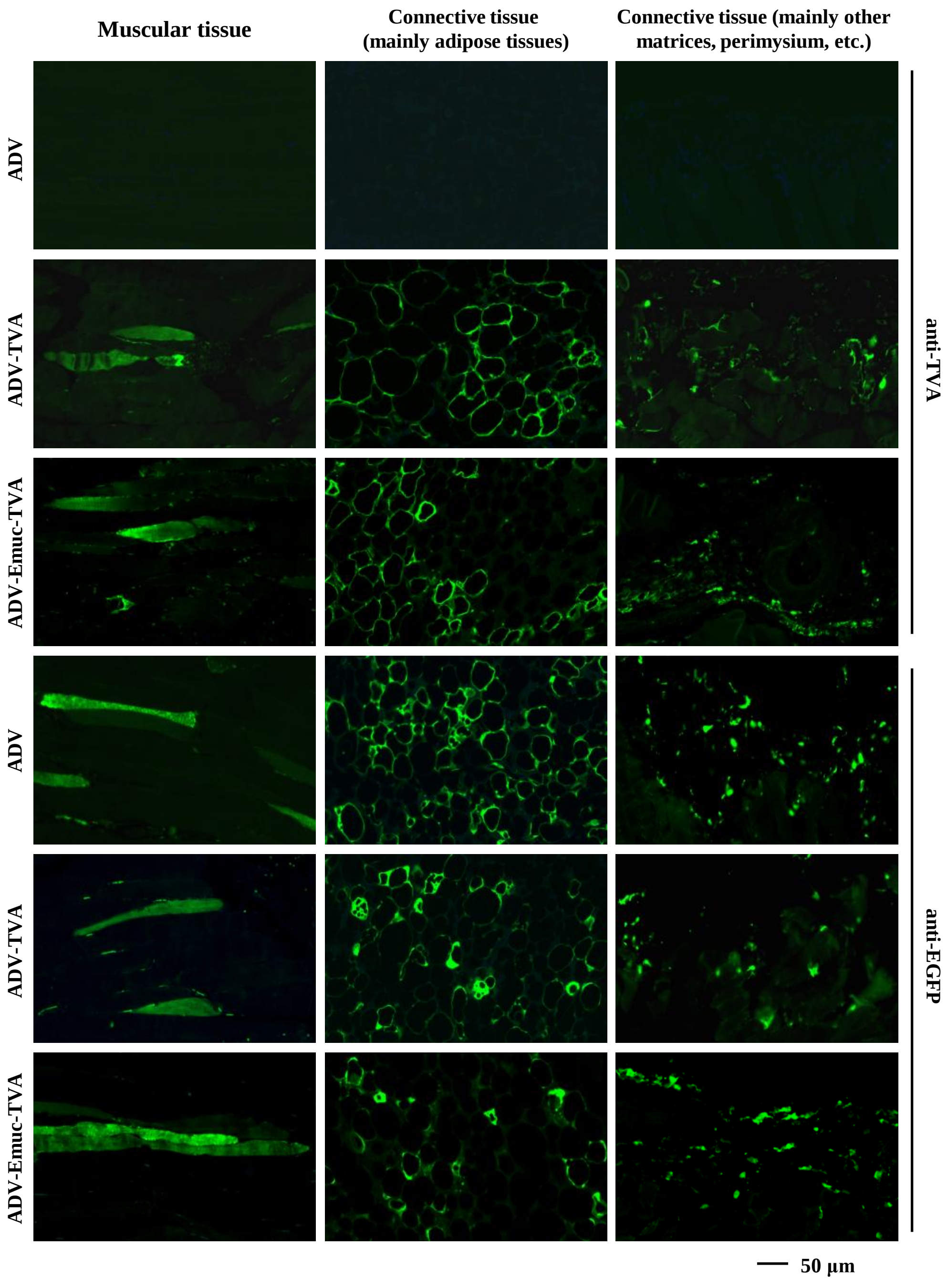

Supplementary Figure S2. Virus transduction and gene expression in tissues. Paraffin embedded tissues were sectioned prior to IFA. Virus infection and gene expression were analyzed by IFA using the anti-TVA or anti-EGFP antibodies, respectively. The adenoviral vectors could infect multiple cell types and implement exogenous gene expression in muscular tissues and connective tissues.

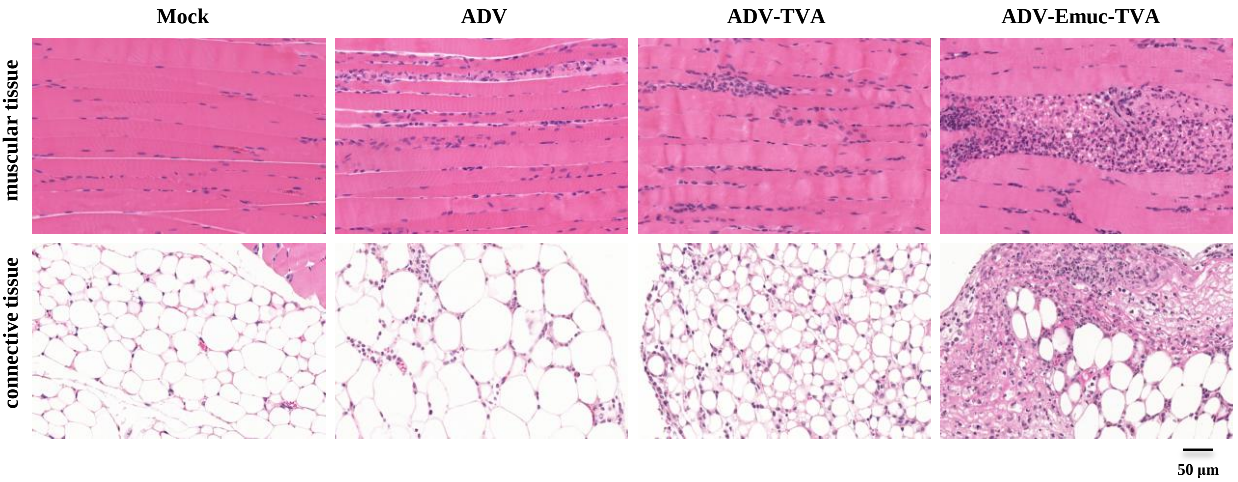

Supplementary Figure S3. Histopathological analyses of the skeletal muscles. Sections of paraffin embedded samples (48 hpi) were stained with H&E for histopathological analyses. ADV-Emuc-TVA infection causes severe inflammation and tissue injury, while the control viruses ADV and ADV-TVA induce mild inflammatory cell infiltration in comparison. See also Figure 1H.

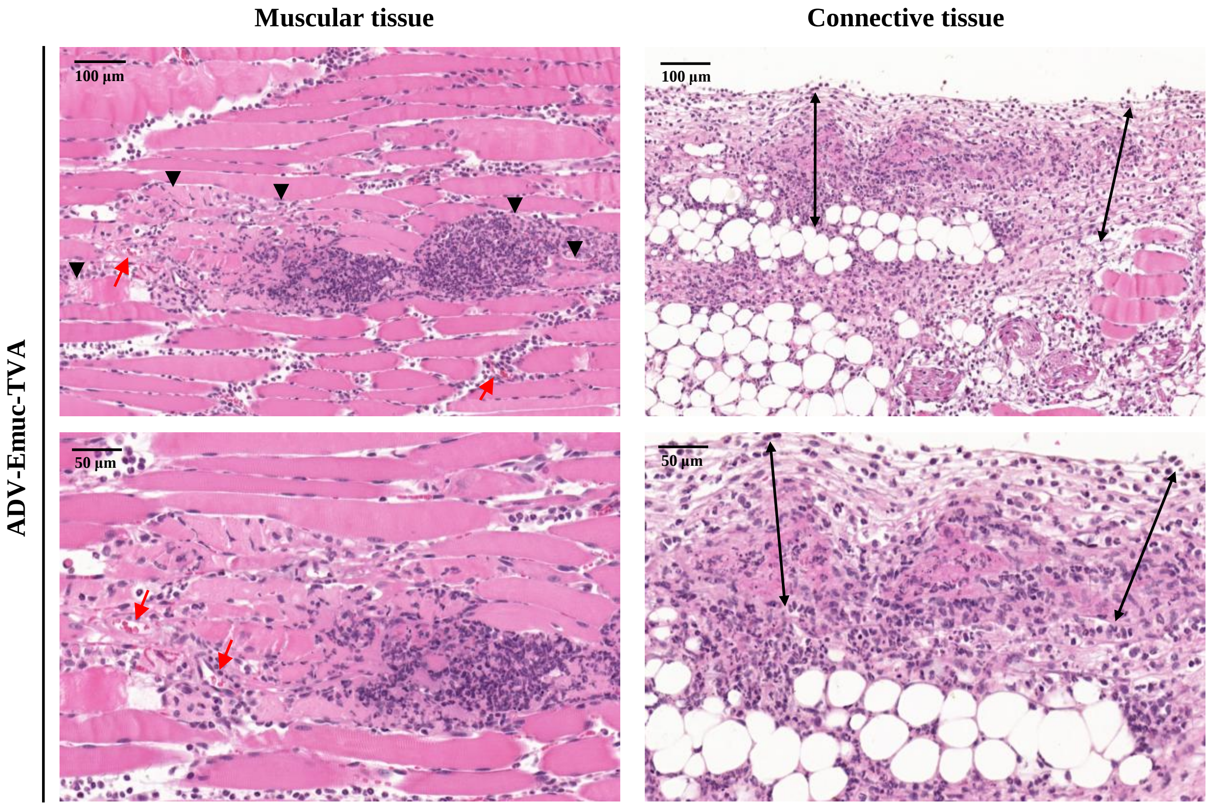

Supplementary Figure S4. Emuc-induced histopathological changes. ADV-Emuc-TVA transduced tissues were closely analyzed by H&E staining. Massive inflammatory cell infiltration was remarkable in both the muscle and connective tissues and local inflammatory cell necrosis could be observed. Disintegration and necrosis of muscle fibers are obvious as representatively indicated by several solid triangles. Red arrows indicate the congested and expanded blood capillaries; hyperplasia of connective tissues accompanied with the inflammatory cell infiltration was indicated by double-headed arrows. Locally enlarged images of the upper panel are shown on the lower panel.

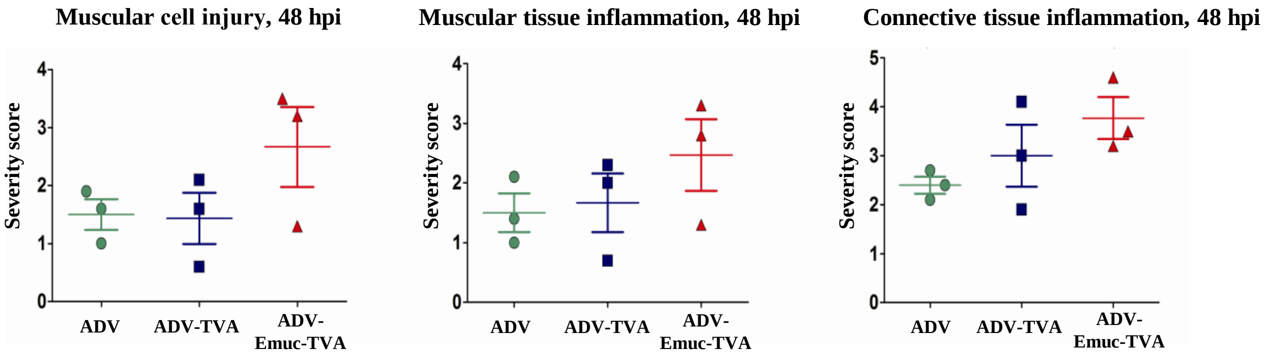

Supplementary Figure S5. Quantification of pathological changes present in tissues. The pathological change degrees of the indicated sample groups (48 hpi) were scored as described in Figure 1J (0 = normal; 1 = minimal change; 2 = mild change; 3 = moderate change; 4 = marked change; 5 = severe change). Graphs show mean ± Standard Deviation, $n = 3$.

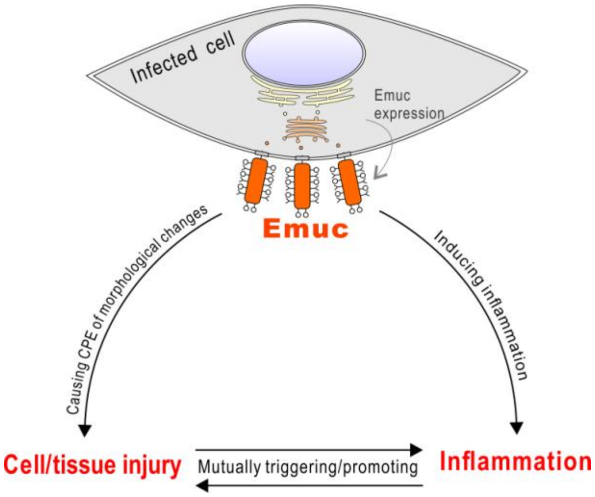

Supplementary Figure S6. Model for Emuc pathologic process. Emuc expression can result in remarkable acute inflammation and tissue damage. On the one hand, Emuc surface expression has the potentiality to cause the CPE of morphological changes as suggested in vitro, which may directly contributes to the cell and tissue injury mediated by Emuc in vivo. On the other hand, Emuc induces significant tissue inflammation, which can be a pivotal indirect cause of the cell and tissue injury. Moreover, cell and tissue injury in turn can also induce inflammatory reactions, and i.e., the cell and tissue injury and inflammation can trigger and aggravate each other under the physiological conditions. These effects may combinedly contribute to the Emuc pathologic process in vivo.
