# Supplementary material for: Ebola virus mucin-like glycoprotein (Emuc) induces remarkable acute inflammation and tissue injury: evidence for Emuc pathogenicity in vivo
Source: Protein Cell. 2017 Sep 27;9(4):389–93. doi: 10.1007/s13238-017-0471-x (PMC5876185; doi:10.1007/s13238-017-0471-x)
Supplement: Supplementary file 2 — Supplementary material 2 (PDF 293 kb) [file 13238_2017_471_MOESM2_ESM.pdf]

Supplementary Materials and Methods

Plasmids and cells.

The cDNA encoding the Ebola virus (EBOV; Zaire/1976/Mayinga, GenBank: AF086833.2) mucin-like region (Emuc) from the full-length glycoprotein (amino acids 312–462) was obtained by gene synthesis (BGI, China). To achieve the normal modification and localization of Emuc in accordance with the context of viral infection, Emuc was cloned into the transmembrane TVA (between the amino acid residues 77 and 78; accession number: L22753) as previously described [1]. The TVA is a small cell membrane glycoprotein which can serve as the membrane-spanning receptor for subgroup A avian sarcoma and leukosis virus [2]. Construction of adenovirus vectors with E1 and E3 deleted (ΔE1/ΔE3) was carried out as described previously [3]. Briefly, the Emuc-TVA construct or TVA alone was subcloned into the plasmid pAdT of the adenovirus generation system in which an EGFP expression cassette has been inserted as a reporter for conveniently monitoring the subsequent gene expression, recombinant virus preparation, and transduction. pAdT and the constructed donor plasmids encoding TVA or Emuc-TVA (named pAdT-TVA or pAdT-Emuc-TVA, respectively) were then linearized with restriction enzyme *Pme* I and co-transformed together with the viral DNA (ΔE1/ΔE3) plasmid into *Escherichia coli* strain BJ5183 by electroporation for homologous recombination. All the cloned plasmids and recombinants were verified by antibiotic resistance screening, restriction enzyme analysis, PCR, and/or sequencing.

Human embryonic kidney (HEK)293, HEK293A, and African green monkey

kidney cells (Vero) were purchased from American Type Culture Collection (ATCC) and cultured in Dulbecco's modified Eagle's medium (DMEM) supplemented with 10% fetal bovine serum (FBS) (GIBCO) at 37℃ in a 5% CO2 atmosphere.

Transfection and generation of recombinant adenoviruses.

The recombinant plasmids were highly purified by Endofree Plasmid Kit (Qiagen) and cleaved with restriction enzyme *Pac* I to expose the ITR (inverted terminal repeats). Using Lipofectamine 2000 (Invitrogen) according to the manufacturer's instructions, the linearized plasmids were then transfected into HEK293 cells in which adenoviral E1 functions can be complemented. Transfection efficiency and viral spread could be monitored by EGFP expression, and recombinant viruses were harvested by multiple rounds of freezing and thawing. The control adenovirus expressing EGFP alone and the recombinant adenoviruses encoding EGFP together with TVA or Emuc-TVA were named ADV, ADV-TVA, and ADV-Emuc-TVA, respectively.

Viral amplification and purification.

Recombinant adenoviruses were amplified for large-scale production with 3×10^8 HEK293A cells in plates. Cells were infected with viruses at a multiplicity of infection (MOI) of 5. Floating and adherent cells were harvested at 72 hours post infection (hpi) and resuspended in DMEM supplemented with 5% FBS. After freezing and thawing, obtained viruses were purified by standard two-step CsCl gradient ultracentrifugation (Stratagene, La Jolla, CA), and subsequently desalted and dissolved into a storage buffer (10 mM Tris, pH 8.0, 2 mM $MgCl_2$ and 4% sucrose).

Purified viruses were stored as single use aliquots at -80℃. Virus titers were determined by tissue culture infectious dose 50 (TCID50) method using 96-well plates.

Antibodies and fluorescent dyes.

Rabbit polyclonal antibodies to Emuc, TVA, or EGFP were raised against the target proteins produced from *Escherichia coli*. The secondary antibody for Western blot was horseradish peroxidase (HRP)-conjugated goat anti-rabbit IgG (Abcam). The Alexa 488- or 555-conjugated goat anti-rabbit IgG secondary antibodies (Abcam) were used for immunofluorescence assay. Hoechst 33258 (Beyotime) was used for the nucleus staining. The PE-conjugated anti-human CD29 (BD Bioscience) and PE-Cy5-conjugated anti-HLA-I (eBioscience) antibodies used to respectively detect the cell surface β1-integrin and HLA-I by flow cytometry were purchased from the indicated manufacturers.

Western blot analyses.

Cell samples were incubated with the lysis buffer (pH7.4, 150 mM NaCl, 1mM EDTA, 1% Triton X-100) supplemented with a cocktail protease inhibitor (Roche) at 4℃ for 30 minutes. Following centrifugation (13000 × g) at 4℃ for 10 minutes, the supernatants of the cell lysates were mixed with the sodium dodecyl sulfate (SDS) sample buffer (25% glycerol, 2.5% SDS, 125 mM Tris, pH6.8, 125 mM dithiothreitol, 0.25% bromophenol blue) and boiled for 10 minutes. Proteins were then separated in 12% SDS polyacrylamide gels and transferred onto polyvinylidene difluoride (PVDF) membranes (Millipore) at a constant current of 110 mA. After blocking with 5%

skimmed milk in Tris-buffered saline-Tween 20 (TBST), the PVDF membranes were probed with the anti-Emuc or anti-TVA primary antibodies and then the HRP-conjugated secondary antibody in 1% skimmed milk-TBST. Protein signals were detected by an enhanced chemiluminescence (ECL) kit (Thermo Fisher Scientific).

Immunofluorescence assays (IFA)

Immunofluorescent staining of cultured adherent cells was performed as previous described [4, 5]. Briefly, cells infected with ADV-Emuc-TVA, ADV-TVA, or ADV at a MOI of 1 were fixed by 4% paraformaldehyde (PFA) at 24 hpi. Unless otherwise specified, cells were then permeabilized by 0.5% Triton X-100 in PBS. After blocking with 2.5% bovine serum albumin (BSA) (Biosharp) and 2.5% normal goat serum (Jackson ImmunoResearch) in PBS, cells were incubated with the anti-Emuc or anti-TVA primary antibodies for 2 hours and then stained with the Alexa 555-conjugated secondary antibody (Abcam) for 1 hour at room temperature. Nuclei were stained with the Hoechst 33258 dye at room temperature for 5 minutes. Images were acquired by a Nikon Ti confocal microscope combined with the Image J software (NIH).

Flow cytometry

HEK293A cells were infected with ADV-Emuc-TVA, ADV-TVA, or ADV at a MOI of 3. At 36 hpi, adherent cells were detached with PBS containing 0.5 mM EDTA and pooled with the harvested floating cells for the following surface immunofluorescent staining of live cells. After washing with 1% BSA-PBS, the

collections of the floating and adherent cells were resuspended and incubated with 5% BSA-PBS at 4℃ for blocking. Aliquot cell samples were incubated with the PE-conjugated anti-human CD29 (β1-integrin) or PE-Cy5-conjugated anti-human HLA-I antibodies at 4℃ for 1 hour, followed by washing. The cell samples were then assayed by a BD Flow Cytometer (LSR Fortessa). For each sample, 10,000 events in the live cell gate drawn on forward and side scatter were analyzed. Data were analyzed by FlowJo software (Tree Star, Inc.). Statistical analyses were performed with GraphPad Prism version 5 (GraphPad Software Inc., San Diego, CA).

Animal experiments

BALB/c mice (n = 3-6, 6-7 week-old females, weight about 18-20 g) were divided to 4 groups randomly. The mice were infected with the recombinant adenoviral vectors (1×10^8 PFU in 50 μl storage buffer) or mock infected with the buffer of 50 μl by intramuscular injection (i.m.) to the great adductor muscle at inner side of the hind limbs. While one of the hind limbs was injected, the other one served as control. Both legs were shaved 24 hours earlier before injection. Animals were monitored daily for clinical features following the administration. The skeletal muscles were harvested at 48 hpi (n = 3) or 72 hpi (n = 6), respectively, and fixed immediately by 4% PFA for at least 24 hours followed by the detection of EGFP signals in tissues using a PE (CRi) Maestro Imager and the histotomy. The mice which were not subjected to the following histopathological analyses were euthanized when they reached an ethical end point.

Animal ethics statement

The animal experiments were approved by the Institutional Animal Ethical Committee of Wuhan Institute of Virology (WIV), Chinese Academy of Sciences (CAS) (Serial number: WIVA223201401) and conducted in the ABSL-2 containment space in the Central Animal Laboratory of WIV under the guidelines of the Care and Use of Laboratory Animals (the Ministry of Science and Technology, China).

Immunohistofluorescence and histopathology

The fixed specimens were washed by running water for 30 minutes after placing in cassettes. All tissues were processed with a graded series of dehydrating agents. The samples were firstly steeped in a graded ethanol series of 75%, 85%, 95%, 100%, and then processed with xylene to clear the ethanol in certain periods of time depending on the thickness of the tissues. Last, samples were embedded in paraffin and sectioned at 3 μm. Slices were dried for 2 hours at 55℃ prior to immunohistofluorescence assay and hematoxylin and eosin (H&E) staining.

The slices were subjected to deparaffinization routinely before the immunostaining. Then sections were steeped in 3% hydrogen peroxide solution for 30 minutes at room temperature to quench endogenous peroxidase activity. Microwave-based epitope retrieval was performed in citrate buffer (10mM, pH 6.0) at 100℃ for 45 minutes. After blocking in 5% BSA for 2 hours at room temperature, sections were incubated with the anti-TVA or anti-Emuc primary antibodies at 4℃ overnight. The secondary antibody was the Alexa 488-conjugated goat anti-rabbit antibody. Following washing, nuclei were stained with the Hoechst 33258 for 10 minutes at room temperature. Section images were captured by Panoramic MIDI

Scanner (3DHISTECH).

H&E staining was performed by an H&E stain Kit (Nanjing JianCheng Technology). To quantify the histopathological changes, ten unduplicated visual fields of muscle or connective tissues for each sample (at the indicated time points post infection) were chosen, respectively. The degrees of pathological changes in comparison to mock-infected samples were scored by the standard as described in [6] with some modifications: 0 = normal; 1 = minimal change (minute quantity of necrosis or inflammatory cells); 2 = mild change (a small quantity of necrosis or inflammatory cells); 3 = moderate change (modest quantity of necrosis or inflammatory cell infiltration); 4 = marked change (large numbers of necrosis or inflammatory cell infiltration); 5 = severe change (diffuse necrosis or inflammatory cell infiltration; almost no remaining normal tissue). Statistical analyses were performed using the GraphPad Prism software.

References

1 Francica JR, Matukonis MK, Bates P. Requirements for cell rounding and surface protein down-regulation by Ebola virus glycoprotein. *Virology* 2009; **383**:237-247.

2 Bates P, Young JAT, Varmus HE. A Receptor for Subgroup-a Rous-Sarcoma Virus Is Related to the Low-Density-Lipoprotein Receptor. *Cell* 1993; **74**:1043-1051.

3 He TC, Zhou SB, da Costa LT, Yu J, Kinzler KW, Vogelstein B. A simplified system for generating recombinant adenoviruses. *Proceedings of the National Academy of Sciences of the United States of America* 1998; **95**:2509-2514.

4 Ning YJ, Wang M, Deng M *et al.* Viral suppression of innate immunity via spatial isolation of

TBK1/IKKepsilon from mitochondrial antiviral platform. *Journal of molecular cell biology* 2014; **6**:324-337.

5 Ning YJ, Feng K, Min YQ *et al.* Disruption of type I interferon signaling by the nonstructural protein of severe fever with thrombocytopenia syndrome virus via the hijacking of STAT2 and STAT1 into inclusion bodies. *Journal of virology* 2015; **89**:4227-4236.

6 Groseth A, Marzi A, Hoenen T *et al.* The Ebola virus glycoprotein contributes to but is not sufficient for virulence in vivo. *PLoS pathogens* 2012; **8**:e1002847.
